# On the SARS-CoV-2 Variants

**DOI:** 10.3390/idr16020024

**Published:** 2024-03-26

**Authors:** Fabio Scarpa, Francesco Branda, Nicola Petrosillo, Massimo Ciccozzi

**Affiliations:** 1 Department of Biomedical Sciences, University of Sassari, Viale San Pietro 43b, 07100 Sassari, Italy; 2 Unit of Medical Statistics and Molecular Epidemiology, Università Campus Bio-Medico di Roma, 00128 Rome, Italy; f.branda@unicampus.it (F.B.); m.ciccozzi@unicampus.it (M.C.); 3 Infection Prevention Control/Infectious Disease Service, Fondazione Policlinico Universitario Campus Bio-Medico, 00127 Rome, Italy

**Keywords:** SARS-CoV-2, SARS-CoV-2 variants, SARS-CoV-2 evolution, pandemic, epidemic

## Abstract

The evolutionary dynamics of viruses, particularly exemplified by SARS-CoV-2 during the ongoing COVID-19 pandemic, underscore the intricate interplay between genetics, host adaptation, and viral spread. This paper delves into the genetic evolution of SARS-CoV-2, emphasizing the implications of viral variants on global health. Initially emerging from the Wuhan-Hu-1 lineage, SARS-CoV-2 rapidly diversified into numerous variants, each characterized by distinct mutations in the spike protein and other genomic regions. Notable variants such as B.1.1.7 (α), B.1.351 (β), P.1 (γ), B.1.617.2 (δ), and the Omicron variant have garnered significant attention due to their heightened transmissibility and immune evasion capabilities. In particular, the Omicron variant has presented a myriad of subvariants, raising concerns about its potential impact on public health. Despite the emergence of numerous variants, the vast majority have exhibited limited expansion capabilities and have not posed significant threats akin to early pandemic strains. Continued genomic surveillance is imperative to identify emerging variants of concern promptly. While genetic adaptation is intrinsic to viral evolution, effective public health responses must be grounded in empirical evidence to navigate the evolving landscape of the pandemic with resilience and precision.

## 1. Introduction

When discussing evolution, genetics, and host adaptation, the focus inevitably turns to viruses, intricate biological entities shaped by Darwinian natural selection. Viruses in general serve as an excellent model for exploring these processes due to their global presence and constant mutation, resulting in a notably high evolutionary rate.

In this context, it is crucial to note that over the past years of the SARS-CoV-2 pandemic, the world has witnessed the potential dangers posed by the dynamics of evolution and genetics.

The Nobel laureate Sir Peter Medawar, in his acceptance speech, remarked that “no virus is known to do good,” encapsulating the perception of viruses as carriers of bad news wrapped in protein. Since the inception of the global pandemic, a myriad of viral variants has arisen, underscoring the remarkable genetic diversity inherent in the virus responsible for the outbreak. This proliferation of variants can be attributed to the heightened variability exhibited by the virus, coupled with its extraordinary mutational capacity. The SARS-CoV-2 virus, causative agent of COVID-19, has demonstrated an exceptional ability to undergo genetic changes, resulting in the continuous emergence of distinct viral lineages [1].

The virus’s genetic plasticity is a consequence of various factors, including selective pressures imposed by host immune responses, the expansive population of susceptible hosts, and the intrinsic error-prone nature of its replication machinery [2]. These factors collectively contribute to the incessant evolution of the virus, giving rise to an extensive repertoire of genetic variants. The identification and characterization of these variants have become integral components of ongoing epidemiological surveillance and research efforts [1].

## 2. First Lineages and First Variants

The genome of SARS-CoV-2 exhibits approximately 82% sequence similarity with SARS-CoV and MERS-CoV, and over 90% sequence identity for crucial enzymes and structural proteins [3]. Structurally, SARS-CoV-2 comprises four structural proteins: spike (S), envelope (E), membrane (M), and nucleocapsid (N) proteins. These proteins demonstrate high sequence resemblance to their counterparts in SARS-CoV and MERS-CoV [4]. Notably, the SARS-CoV-2 genome is noted for its instability at elevated temperatures due to a highly enriched A+U content (62%) and reduced G+C content (38%), comparable to the hCoV-OC43 genome (63% A+U and 37% G+C) and the hCoV-NL63 genome (66% A+U and 34% G+C) [5]. The initial Wuhan-Hu-1 lineage identified in late 2019 displayed a high level of entropy with a rapid evolutionary rate, indicative of the typical trend preceding a surge in population size and, consequently, contagion. Consequently, as observed in the early expansion of viruses, the genome evolution was initially rapid, driven by the need for swift adaptation to the new host. Indeed, the evolutionary rate of the SARS-CoV-2 Wuhan-Hu-1 variant reached about 6.58 × 10^−3^ subs/site/year [6].

Following the initial expansion, several variants have been identified based on mutations in the S1 subunit of the spike (S) protein, particularly in its receptor-binding domain (RBD).

The SARS-CoV-2 B.1.1.7 (α), also known as the UK variant, which rapidly spread across various European countries to become globally dominant, carried a mutation at position 501, where asparagine was replaced with tyrosine, and there was a deletion of two amino acids at position 69–70 of the spike protein sequence. The N501Y mutation impacted the conformation of the receptor-binding domain, resulting in enhanced transmission capabilities [7]. Phylodynamic reconstruction indicated a high level of variability with an exponential growth of lineages until May–June 2021 [8,9]. Subsequent Variants of Concern (VOCs), such as B.1.351 (β) and P.1 (γ), first identified in South Africa and Brazil, respectively [10], were primarily characterized by mutations E484K and N501Y [11]. They exhibited heightened contagion and resistance to neutralization by antibodies from convalescent and vaccine-recipient individuals, raising concerns, including issues related to misidentification due to shared mutations [12].

The B.1.617.2 (δ) variant, first identified in India, presented two amino acid changes in the spike protein sequence (L452R and P681R) compared to previous variants [13]. These changes were critical for binding with ACE2 and raised specific concerns due to an increased positive electrostatic potential resulting from three amino acid changes, shifting from negative or neutral to a clearly positive charge [14]. There is likely a correlation between the heightened positive electrostatic potential and affinity for the ACE2 receptor in the B.1.617 variant, particularly in B.1.617.2. ACE2, with surface patches of negative electrostatic potential, likely experiencing increased tropism of the virus Spike due to the higher positive potential of the RBD, enhancing overall interaction affinity. This condition was supported by a phylogenomic overview indicating that the B.1.617 variant and its sublineages (especially B.1.617.2) exhibited high levels of diversity and expansion capabilities initially (until the second half of 2021). Subsequently, the evolutionary rate began to slow down as the variant had “exhausted its capacity to adapt”, becoming endemic. 

## 3. The Last Variants

The most recent Variant of Concern (VOC) that marked a significant departure in terms of the genomic composition of the virus was the Omicron variant. Initially identified in South Africa in November 2021, the SARS-CoV-2 Omicron variant exhibited a considerable number of mutations in the spike protein, raising uncertainties about its transmissibility and lethality compared to previous variants. Rapidly becoming the dominant worldwide variant, the Omicron strain gave rise to numerous subvariants, creating a series of new lineages and sublineages that sparked various concerns [15]. In comparison to earlier variants, the Omicron variants of SARS-CoV-2 showed a mutation rate in the receptor-binding motif (RBM) that was 5.5 to 11 times higher [16]. The spread of the B.1.1.529 variant was facilitated by its expansion during colder months, known for favoring the transmission of respiratory viruses. Notably, B.1.1.529 exhibited 32 new mutations in the spike protein, a significantly higher number than any other VOC [17]. This variant displayed a marked increase in the positive electrostatic potential at the RBD interface with ACE2 [17]. While some changes occurred, such as the removal of two salt bridges and the formation of two others, there were no drastic alterations observed. Global phylogenomic reconstruction indicated a high genetic differentiation between B.1.1.529 and previous variants, and within the Omicron clade, a fast mutation rate reflecting rapid evolution. This, coupled with the direct correlation between electrostatic potential and receptor affinity, contributed to the high transmissibility of the Omicron VOC, as expected. The altered surface electrostatic potential in RBD likely aided its expansion by modifying interactions with other macromolecules such as antibodies [17]. Consequently, the Omicron variant presented several subvariants and sublineages with varying expansion capabilities, causing concerns for several years. In the first half of 2022, the Omicron SARS-CoV-2 BA.5 lineage emerged as the dominant and most widespread variant in circulation. Despite a prolonged period of high case numbers, BA.5 demonstrated limited expansion capabilities, showing relatively low genetic variability after its initial spread and reaching a plateau on 6 June 2022 with an evolutionary rate of about 7 × 10^−4^ subs/site/year [18], notably slower (about ten times) than the evolutionary rate of the first SARS-CoV-2 lineage Wuhan-Hu-1 variant [6], making it ten times slower than BA.5. Mid-2022 saw concerns about the Centaurus variant (BA.2.75), leading to a temporary increase in infections. Genetic and structural data indicated that BA.2.75 evolved even more slowly than previous variants, suggesting it would not become dominant [18], and, indeed, it did not. Like BA.5, this variant exhibited a lower evolutionary rate (1.6 × 10^−4^ subs/site/year), preventing further expansion of the viral population size, with the peak phase beginning around 23 June 2022, and no subsequent increase [18]. All subsequent variants causing concerns and requiring monitoring followed a pattern of vicariance, taking turns in succession without becoming truly worrisome. For example, the BQ.1 variant, nicknamed *Cerberus*, descended from BA.5 [19] and showed a very similar evolutionary rate of 7.6 × 10^−4^ subs/site/year [20]. It replaced its direct ancestor in terms of diffusion, peaking around 3 September 2022. Afterward, its worldwide distribution stopped, entering a plateau phase with no further expansion [20]. The last two variants causing concern in 2022 were XBB and XBB.1.5, nicknamed *Gryphon* and *Kraken*, respectively [21,22]. The recombinant XBB, with an evolutionary rate of 7.6 × 10^−5^ subs/site/year, peaked around 6 October 2022, and was initially present mainly in South Asia where the recombination event likely occurred [21]. XBB spread relatively slowly and never experienced significant population size or contagiousness expansion. Its first significant descendant, XBB.1.5, displayed an evolutionary rate of 6.9 × 10^−4^ subs/site/year, peaking around 24 November 2022 [22], remaining confined to selected regional areas of the USA [23]. Similarly, the SARS-CoV-2 BF.7 lineage, one of the most recent variants, showed limited diffusion to the Asian region, causing considerable concerns at the beginning of 2023 due to a resurgence in COVID-19 cases [24]. In this case, the population size experienced minor fluctuations over time, reaching its peak for the last time around 14 December 2022. The evolutionary rate of 5.62 × 10^−4^ subs/site/year further reflects low genetic variability and limited capacity for significant demographic expansion [24]. All the variants exhibit a phylogenomic reconstruction with molecular features typical of an evolutionary dead-end branch with no further significant descendants [18,20,21,22,24]. They have been characterized by localized distribution and/or limited demographic expansion. Structurally, it is interesting to note an apparent trend toward an increase in positive electrostatic potential from the original virus strain through the B.1.617.2 variants and its descendants up to the most recent Omicron variant. Several mutations present in the RBD sequence have been shared by many SARS-CoV-2 Omicron variants.

## 4. The Year 2023

In a similar fashion, the year 2023 also witnessed the emergence of numerous variants, each with varying levels of concern. However, none of them truly posed a substantial threat, even though their appearance each time stirred considerable caution and anxiety. These variants, while causing heightened vigilance and concern with each occurrence, did not manifest into a tangible and significant problem. The ongoing monitoring and study of these variants underscored the dynamic nature of the virus, emphasizing the need for continued research and surveillance to ensure effective public health responses. Despite the fluctuations in the appearance of these variants, their impact did not escalate into a concrete and widespread challenge throughout the year. The SARS-CoV-2 EG.5 variant appeared in early 2023 [25] and, after causing the usual worries, genome-based surveys demonstrate that EG.5 and descendants presented a restricted ability for substantial population growth, aligning with the evolutionary trajectory of the recent sub-variant that initially caused apprehension [26]. In addition, evolutionary rates observed were roughly tenfold lower than that of the original SARS-CoV-2 Wuhan-Hu-1 strain. The recombinant XBF attracted attention in the first half of 2023, such as the variant XBB.1.16. In both cases, genomic and structural analyses indicated that, despite carrying multiple noteworthy spike mutations, there was no evidence suggesting heightened risk or substantial expansion capability [27]. The peak in viral population size had been reached in several months, during which the lineages circulated almost unnoticed, showing features different from the epidemiologically perilous lineage observed at the onset of the pandemic. The most recent significant variant identified is SARS-CoV-2 BA.2.86, also known as *Pirola* [28]. Concerns surrounding BA.2.86 arise from numerous new mutations detected when comparing it to its putative ancestor BA.2. Specifically, spike mutations have garnered attention as they potentially pose theoretical threats, such as P681R and F486P. The P681R mutation notably played a pivotal role in the spread of Delta variants [29]. Theoretically, reverting the P681R mutation to its original form (wild-type P681) significantly reduces replication; hence, the pathogenicity of BA.2.86 is theoretically higher than BA.2. The P681R mutation appears to enhance the cleavage of the spike protein into S1 and S2, potentially enhancing viral cell entry [29]. Studies on the Delta variant demonstrated that the P681R-bearing virus exhibits higher pathogenicity compared to its parental virus [30]. The F486P mutation replaces the original Wuhan Phe (also found in BA.2) with a Pro. A similar mutation was identified in XBB.1.5 [22], where structural analyses indicated that replacing the original F486 with Ser in XBB and XBB.1 disrupts the interface’s stability [22], while introducing Pro at position 486 appears to partially restore the contribution of free energy to interface stability [22]. The conserved F486 residue in BA.2 can form Van der Waals interactions with amino acids located within a hydrophobic pocket on the ACE2 receptor. The introduction of Pro486 potentially enhances the rigidity of the loop in which it is situated. It could be suggested that the somewhat improved stability brought about by Pro might also involve an entropic aspect. Specifically, reducing the flexibility of the loop could lead to a decrease in its entropy loss during the formation of the complex, as previously demonstrated for XBB.1.5 [22]. However, it is important to emphasize that the theoretical potential of a single mutation should only be used to determine whether it warrants attention or not, as its actual impact in a specific variant is also influenced by its interaction with other mutations. In fact, in several cases, it has been observed that mutations deemed dangerous in previous variants did not necessarily have particularly perilous effects in more recent variants.

Currently (as of mid-February 2024), a new emergent lineage has been identified in South Africa [31]. This lineage, labeled SARS-CoV-2 BA.2.87.1 and characterized by over 100 mutations, is likely the most divergent lineage identified this year. The nine isolated genomes were collected between mid-September and mid-November 2023. Like previous variants, this lineage requires particular attention, especially due to the high number of new mutations [31].

## 5. Potential Factors Related to the Emergence of Genetic Variants

The described variants arise due to a complex interplay of factors, including selective pressures exerted by host immune responses, spontaneous mutations occurring during viral replication, events of inter-species transmission, transmission dynamics within human populations, and environmental influences [32]. 

Indeed, the emergence of variants can occur in response to a myriad of selective pressures exerted by the host immune system, population dynamics, and public health interventions. The host immune response represents a significant force driving viral evolution [33]. As the virus interacts with the host immune system, mutations that confer a selective advantage, such as enhanced immune evasion or increased replication efficiency, may arise and become prevalent within the viral population [34]. Moreover, the landscape of susceptible hosts within a population undergoes dynamic changes due to natural infection, vaccination campaigns, and other factors. This dynamic interplay between host immunity and viral adaptation influences the emergence and spread of genetic variants. Furthermore, public health measures implemented to control the spread of COVID-19, such as social distancing, mask mandates, and vaccination efforts, introduce additional selective pressures on the virus. Variants that possess traits enabling them to evade these interventions, such as increased transmissibility or immune escape, are typically more likely to thrive and propagate. Consequently, the ongoing interplay between host immunity, population dynamics, and public health interventions shapes the genetic diversity of SARS-CoV-2, highlighting the intricate relationship between viral evolution and the epidemiology of COVID-19. Understanding these selective pressures is crucial for predicting the emergence of novel variants and developing effective strategies to control the pandemic. On the issue related to spontaneous mutations, it should be pointed that the genetic makeup of SARS-CoV-2 is continually subjected to mutations as the virus replicates within host cells [35]. These mutations arise due to errors in the viral replication process, as well as selective pressures exerted by the host immune system or other environmental factors. While many mutations have little to no impact on the virus’s biology, some can lead to significant changes in viral properties. For example, mutations may occur in regions of the viral genome responsible for viral entry into host cells, replication machinery, or immune evasion mechanisms. Occasionally, these mutations confer a selective advantage to the virus, allowing it to replicate more efficiently, evade host immune responses, or enhance transmission [36]. Such advantageous mutations may become fixed within the viral population over time, leading to the emergence of new genetic variants. Moreover, the accumulation of mutations over successive replication cycles can contribute to the genetic diversity of SARS-CoV-2, allowing the virus to explore a wide range of phenotypic traits. Understanding the mechanisms underlying spontaneous mutations and their role in shaping viral evolution is crucial for predicting the emergence of novel variants and developing targeted interventions to control their spread. The further significant factor potentially contributing to the emergence of genetic variants of SARS-CoV-2 is the occurrence of inter-species transmission events, particularly from animals to humans [37]. These events, known as zoonotic spillover, can introduce novel genetic material into the human viral pool, leading to the generation of new variants with unique adaptive traits. Zoonotic spillover events may occur when humans come into close contact with animals harboring related coronaviruses, facilitating the transfer of the virus from its natural reservoir to humans [38]. Such interactions can occur in various settings, including wildlife markets, farms, or domestic settings where humans interact closely with animals. Once the virus is transmitted to humans, it may undergo further adaptation and evolution within the human host population, leading to the emergence of variants with altered transmission dynamics or pathogenicity [37]. Additionally, human activities such as deforestation, urbanization, and agricultural practices can disrupt natural ecosystems, increasing the frequency of interactions between humans and wildlife and facilitating the spillover of zoonotic pathogens. Understanding the dynamics of inter-species transmission and the factors influencing these events is crucial for predicting and mitigating future zoonotic outbreaks. Moreover, comprehensive surveillance programs aimed at monitoring viral diversity in both human and animal populations are essential for early detection and containment of emerging zoonotic threats. By addressing the root causes of zoonotic spillover and enhancing our ability to detect and respond to emerging infectious diseases, we can better prepare for and prevent future pandemics. On the other hand, the transmission dynamics of SARS-CoV-2 within human populations also play a critical role in shaping the genetic diversity and evolution of the virus [39]. Factors such as population density, social behavior, and healthcare infrastructure influence the spread and diversification of viral variants. High population density, coupled with close interpersonal contact, facilitates the transmission of the virus between individuals, providing ample opportunities for viral replication and mutation. Social behaviors, such as travel patterns, adherence to public health guidelines, and vaccination rates, also impact transmission dynamics. Variants that are more transmissible or better adapted to spread in specific social contexts may become dominant within a population, leading to shifts in the genetic composition of circulating viruses. Additionally, healthcare infrastructure, including diagnostic testing capacity, healthcare access, and vaccination distribution, can affect transmission dynamics by influencing the detection and control of viral spread [40]. Variants that evade detection or spread more rapidly within healthcare settings can contribute to localized outbreaks and fuel the emergence of new variants. Understanding the complex interplay between transmission dynamics and viral evolution is crucial for implementing effective control measures to mitigate the spread of COVID-19. Public health interventions, such as vaccination campaigns, testing strategies, and targeted quarantine measures, should be tailored to address the specific transmission dynamics of circulating variants and prevent further dissemination of the virus [40]. Furthermore, ongoing surveillance efforts to monitor changes in transmission patterns and identify emerging variants are essential for guiding public health responses and mitigating the impact of the pandemic. By incorporating transmission dynamics into our understanding of viral evolution, we can develop more effective strategies to control the spread of SARS-CoV-2 and prevent future outbreaks. Environmental selection pressures, such as temperature, humidity, and geographic location, can influence the survival, transmission, and spread of a virus in general. For example, studies have suggested that SARS-CoV-2 may exhibit seasonal variation in transmission rates, with higher transmission occurring in colder, drier climates compared to warmer, more humid environments [41]. These environmental conditions can directly impact viral stability and persistence in the environment, as well as human behavior and social interactions, which in turn influence transmission dynamics. Variants that are better adapted to specific environmental conditions may have a competitive advantage, leading to their preferential spread in certain regions or seasons [42]. Additionally, environmental factors may indirectly influence viral evolution by shaping the distribution and abundance of host species, including reservoirs and intermediate hosts. Changes in land use, urbanization, and agricultural practices can alter natural ecosystems, increasing the frequency of interactions between humans, animals, and the environment and facilitating the spillover of zoonotic pathogens. Understanding the role of environmental selection in driving viral evolution is essential for predicting the emergence and spread of new variants and implementing targeted interventions to mitigate the impact of the pandemic. By incorporating environmental factors into our surveillance and control efforts, we can better understand the complex interplay between human activities, environmental change, and infectious disease emergence, ultimately reducing the risk of future pandemics.

## 6. Conclusions

For all of the described reasons, continued and uninterrupted genome-based monitoring is crucial to identify, as soon as possible, variants that really can represent a threat. In general, the new characteristics of an emerging variant are typically linked to advantages that stem from genetic drift, enabling the virus to continually adapt to the host without necessarily conferring a direct fitness advantage. It is crucial not to interpret this as a justification for complacency in the face of the pandemic. While maintaining vigilance, decisions should be grounded in data, and fear should not cloud judgment. 

In summary, the prolific emergence of SARS-CoV-2 variants underscores the virus’s capacity for genetic adaptation, necessitating a comprehensive and vigilant scientific approach to monitor, analyze, and respond to the evolving landscape of the pandemic.

## Data Availability

Not applicable.
